# All the Pups We Cannot See: Cannibalism Masks Perinatal Death in Laboratory Mouse Breeding but Infanticide Is Rare

**DOI:** 10.3390/ani11082327

**Published:** 2021-08-06

**Authors:** Sophie Brajon, Gabriela Munhoz Morello, Sara Capas-Peneda, Jan Hultgren, Colin Gilbert, Anna Olsson

**Affiliations:** 1 Laboratory Animal Science, IBMC—Instituto de Biologia Molecular e Celular, and i3S—Instituto de Investigação e Inovação em Saúde, Universidade do Porto, Rua Alfredo Allen, 4200-135 Porto, Portugal; sophie.brajon.1@ulaval.ca (S.B.); gabriela.morello@commonhomeofhumanity.org (G.M.M.); sara.capas@ibmc.up.pt (S.C.-P.); 2 Babraham Institute, Babraham, Cambridge CB22 3AT, UK; colin.gilbert@babraham.ac.uk; 3 ICBAS School of Medicine and Biomedical Sciences, University of Porto, Rua Jorge de Viterbo Ferreira 228, 4050-313 Porto, Portugal; 4 The Francis Crick Institute, 1 Midland Road, London NW1 1AT, UK; 5 Department of Animal Environment and Health, Swedish University of Agricultural Sciences, 53223 Skara, Sweden; jan.hultgren@slu.se

**Keywords:** neonatal mortality, husbandry practices, cage inspection, pup-counting method, social environment, cannibalistic behaviour, asynchrony breeding, mouse welfare, 3Rs principle

## Abstract

**Simple Summary:**

Perinatal mortality is a large problem in laboratory mouse breeding. Dead pups are often eaten by the adult mice. Pups cannibalised between birth and first husbandry check are likely not to be accounted for, leading to an underestimation of the number of pups are born and die. This study aimed at understanding to what extent perinatal mortality is underestimated in a breeding facility. The roles of cannibalism and infanticide (killing of a live pup) in pup mortality were also investigated. The results indicated that the standard pup-counting method by daily cage checks led to an underestimation of the number of pups that are born by 35% and those that die by 102% compared to data where daily checks were combined with video observations from which cannibalistic events could be recorded. Cannibalism of dead pups before the first check explained this inaccuracy in death counts, while infanticide was rare. Beyond considerations of animal welfare and ethics, and a conflict with the 3Rs principle, high perinatal mortality means that larger colonies of breeding animals are needed to supply animals in sufficient numbers for research. Paradoxically, the common practice of not disturbing cages around parturition conceals the extent of perinatal mortality but has also constrained the determination of causes of pup death.

**Abstract:**

Perinatal mortality is a major issue in laboratory mouse breeding. We compared a counting method using daily checks (DAILY_CHECK) with a method combining daily checks with detailed video analyses to detect cannibalisms (VIDEO_TRACK) for estimating the number of C57BL/6 pups that were born, that died and that were weaned in 193 litters from trios with (TRIO-OVERLAP) or without (TRIO-NO_OVERLAP) the presence of another litter. Linear mixed models were used at litter level. To understand whether cannibalism was associated with active killing (infanticide), we analysed VIDEO_TRACK recordings of 109 litters from TRIO-OVERLAP, TRIO-NO_OVERLAP or SOLO (single dams). We used Kaplan-Meier method and logistic regression at pup level. For DAILY_CHECK, the mean litter size was 35% smaller than for VIDEO_TRACK (*p* < 0.0001) and the number of dead pups was twice lower (*p* < 0.0001). The risk of pup loss was higher for TRIO-OVERLAP than TRIO-NO_OVERLAP (*p* < 0.0001). A high number of pup losses occurred between birth and the first cage check. Analyses of VIDEO_TRACK data indicated that pups were clearly dead at the start of most of the cannibalism events and infanticide was rare. As most pups die and disappear before the first cage check, many breeding facilities are likely to be unaware of their real rates of mouse pup mortality.

## 1. Introduction

The mouse is the most widely used laboratory species, with 6.20 million animals reported as used in biomedical research or for the creation of new, genetically altered animal lines in the European Union in 2017 [1]. The total number of mice bred is greater, as these statistics do not include animals that die naturally or are culled before being used. Of these, a substantial proportion will have died shortly after birth. Existing literature indicates highly variable pup mortality rates from less than 5% [2] up to 60% or more [3,4] in experimental studies with C57BL/6 mice, whereas our data from historical records analysed in two different facilities in the United Kingdom revealed perinatal mortality rates of 14 and 39%, respectively [5]. Perinatal mortality often includes loss of the entire litter [6,7]. High mortality rates are an animal health and welfare concern and the loss of large numbers of pups violates the 3Rs principle (replacement, reduction, refinement; [8]). In addition, breeding facilities must maintain larger breeding colonies in order to compensate for pup loss, which is costly and adds pressure on limited building and personnel resources.

In recent years, some efforts have been made to evaluate the scale and to comprehend the factors leading to pup and litter loss. Factors related to the genetic background [7,9,10], management routines [2], physical environment [11,12,13,14] and social environment [15,16,17] are known to affect pup survival. In a previous study, we demonstrated that in litters born in a trio cage (one male plus two females) where there is already another litter (i.e., litter overlap) the risk of pup loss is dramatically increased, by 1.8 times [6]. The phenomenon of litter overlap, which is the result of reproductive asynchrony between the two females of the trios, may be common in laboratory mouse breeding. In a retrospective analysis of data records from 34,949 litters, an estimated 55% of these were born into a situation of litter overlap [5].

Accurate estimation of perinatal mortality in practice is a challenge. Adults often cannibalise dead pups, making them uncountable, and perinatal mortality typically occurs within the first few days of life, the period in which many animal facilities avoid inspection of breeding cages to prevent disturbing the animals. This means that cage inspections even 12–24 h after birth may result in an underestimation of perinatal mortality, and cage inspections three days after birth may be totally inadequate in this regard.

The common practice of not inspecting cages around parturition prevents us from understanding and determining the causes of pup death. There is a common belief in breeding facilities that infanticide (the act of killing a newborn) is a major cause of pup mortality. This belief may be based on a conflation of cannibalistic behaviour (the act of eating a dead body) and infanticidal behaviour. Even in the behavioural literature, infanticide is often conflated with cannibalism; for example Garner et al. [18] defined infanticide as “litters born and disappearing, finding dead pups, finding parts of pups, or observing animals killing pups”. Nevertheless, infanticide in laboratory mouse is plausible and could be motivated by increased litter size, reduced food supply or disturbance [19]. Studies from behavioural ecology have described various manipulative tactics in wildlife, including infanticide [20,21], to improve the manipulator’s reproductive success and survival. For instance, infanticide has been shown to be relatively common in species practicing communal nesting where several females nurse their offspring together in a single nest, such as wood and house mice [22,23]. In such wild species, a female may reduce the litter size of another female through infanticide as an exploitation strategy to bias the relative contribution to the communal litter in her own favour and thus increase her fitness [24].

There is little evidence for infanticide in laboratory mice, possibly because females bred together with a male are genetically related and kept in stable conditions [25]. Schmidt et al., [24] reported infanticide in 67 litters from outbred mice derived from inbred strains, but this was inferred from the disappearance of pups between cage inspection without behaviour observations. This technique only allows that pups were cannibalised to be detected, but does not establish whether they were previously killed by an adult. Weber et al. [26] performed detailed video observations of five C57BL/6 females who were housed alone and lost their entire litter before weaning, and they were not able to detect infanticide.

In the present study, we used video recordings from parturition and onwards [6]. Through detailed analysis of these videos, with focus on detecting cannibalism events (and thus dead pup disappearance), we were able to estimate actual litter size at birth. The first objective was to compare these data to breeding colony data, where litter size was estimated by daily cage checks without knowing if pups were lost between birth and the first cage check. The next objective was to evaluate timing of pup mortality and the risk of cannibalism, using detailed analyses of cannibalism events. The third objective was to determine whether pups were already dead when cannibalised or were first killed by an adult. Finally, we aimed to evaluate the impact of social housing on cannibalism and infanticide.

## 2. Materials and Methods

### 2.1. Datasets

The data collection took place at the Biological Support Unit of the Babraham Institute (Cambridge, UK) from March to June 2017 and included two subsamples from the breeding colony of wild-type mice derived from inbred C57BL/6Babr parent stock.

Experimental data were collected by the first author of this paper from a total of 109 litters of mice previously housed in trios, including 31, 43, 26 and 9 litters from parities 2, 3, 4 and 5, respectively. For additional details about the litters and conditions, see Brajon et al. [6]. Gestating females were allocated to a social housing configuration approximately three days before parturition, either in single-housing (SOLO; 54 litters, 472 pups) or group-housing in trios of two females and a male, further divided into trios with (TRIO-OVERLAP; 20 litters, 170 pups) or without (TRIO-NO_OVERLAP; 35 litters, 308 pups) the presence of a sibling litter. A newborn litter was considered as being born in TRIO-OVERLAP cage when another litter was already present in the cage at the time of birth. This sibling litter was thus older but could be born anytime from several minutes to 22 days before the newborn litter.

Breeding-facility data were extracted from the special breeding management software (MCMS, Mouse Colony Management System, Wellcome Sanger Institute, Hinxton, UK) and provided by the Babraham Institute’s breeding facility. Data extracted from the management software were data recorded at the same time, from gestating mice of the same parity range, located in the same room as the experimental data, but they included mice from different litters. This resulted in a total of 138 litters of wild-type mice derived from C57BL/6Babr parent stock, including 51, 45, 24 and 18 litters from parities 2, 3, 4 and 5, respectively. The standard social configuration in the colony was trio, and another litter could be present (i.e., TRIO-OVERLAP; 70 litters) or not (i.e., TRIO-NO_OVERLAP; 68 litters) at the time of detection of the focus litter during the cage check (day 0). The breeding facility dataset contained information on litter identity, adults identities, date of birth, number of pups born, number of pups weaned, and the presence of another litter.

### 2.2. Housing

All mice were housed in individually ventilated cages of transparent polysulphone (Tecniplast GM500 Filter©, Buguggiate, Italy; L × W × H, 391 mm × 199 mm × 160 mm), mounted on digital ventilated holding units (DGM Sealsafe Plus Rack©, Tecniplast, Buguggiate, Italy). Cages were supplied with an average of 48 g of soft wood-flake bedding (Eco-Pure Chips 6 Premium©, Datesand, Manchester, UK) and 7.5 g of white paper rolls (Enrich-n’Nest©, Datesand, Manchester, UK) as nesting material. Room temperature was kept at 20–24 °C and relative humidity at 45–65%. Lights were maintained at a 12:12 h regime with lights switched on gradually from 07:00 and switched off gradually from 19:00, leaving residual low level red light only. The animals were given standard 9.5 mm dry food pellets (CRM (P) Vacuum Pack, Dietex International Ltd., Witham, UK) and sterilised water in automatic drinking valves (Edstrom A160/QD2, Avidity Science LLC, Waterford, WI, USA), both *ad libitum*. The cages of the breeding facility were furnished with a hanging transparent plastic box (Tecniplast Mouse Dual Purpose Pouch Loft, Tecniplast, Buguggiate, Italy) located in the front part of the cage. Because this would have interfered with video recordings, the plastic boxes were replaced by a red polycarbonate tunnel (International Product Supplies Ltd, London, UK; L × Ø, 98.55 mm × 50.80 mm) suspended from the cage lid in the experimental study.

### 2.3. Management Routines

**Figure 1:**
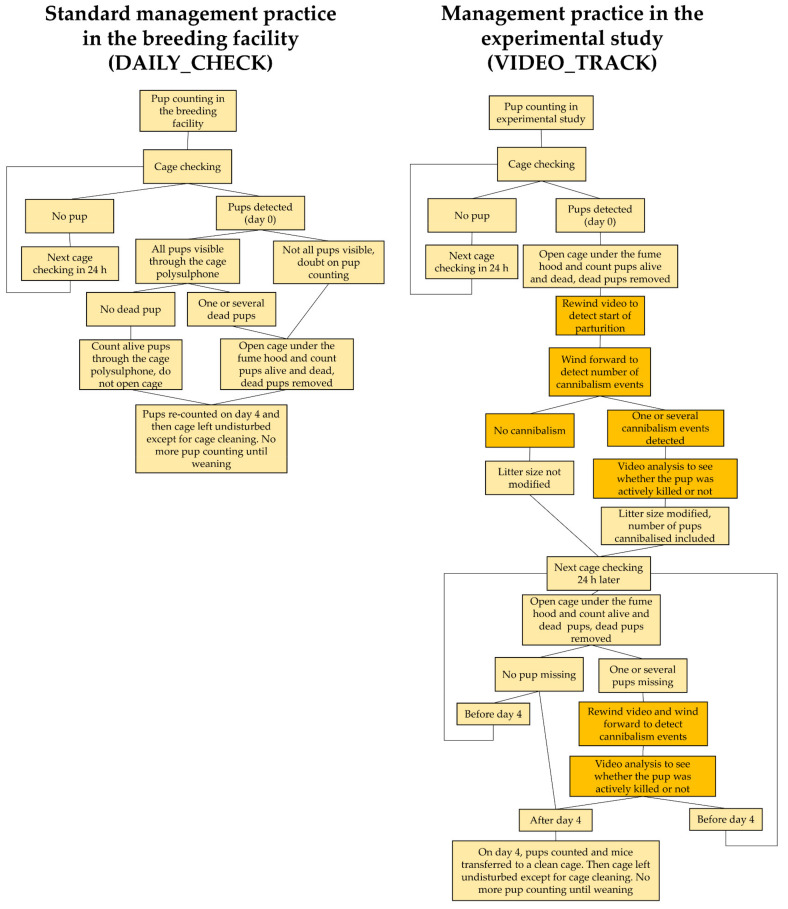
Flowchart describing the different steps of the pup-counting procedure used to determine the litter size in standard management practice at the breeding facility (**left**) and the experimental study (**right**). Orange boxes concern the procedure of video analysis used afterward to detect cannibalism events and adjust litter size post-hoc if needed in the experimental study.

Figure 1 summarises how litter size was determined and cannibalism was detected in the two datasets. The standard management practices of the breeding facility (i.e., DAILY_CHECK counting) involved the daily inspection of cages with periparturient females to identify the day of birth of each litter (day 0), performed by technicians from the breeding facility. This was carried out by removing cages from the rack and counting living and dead pups directly through the cage transparent polysulphone. If the litter was not clearly visible or if dead pups were seen, the cage was opened at the change station under a fume hood for closer examination and dead pups were removed from the cage. The technician disinfected his/her hands with 70% ethanol before opening a cage and touching its inside. When a dam seemed not to have finished parturition or if few pups were present, the pups were counted again the next day. On day 4, all cages were opened and the pups re-counted at the change station under the fume hood. Except for cleaning every two weeks (but not before four days post-birth), the cages were then left undisturbed until weaning on day 21 ± 2 when weaned pups were counted. In the experimental study (i.e., VIDEO_TRACK counting), the cages were inspected by the first author once a day to identify day of birth (day 0), using the same precautions as in the breeding facility, except that cages were systematically opened at the change station once pups born for a more accurate visual inspection. Additionally, living and dead pups were then counted at daily cage inspections until day 4. Pups were touched gently if needed to count them and dead pups were removed. In addition, counting was followed by video analyses to validate and/or rectify litter sizes according to cannibalism events (see Section 2.4). All mice were transferred to a clean cage on day 4. Except for cleaning every two weeks, the cages were then left undisturbed until weaning. The alive pups were weaned and counted on day 21 ± 2. A summary of the study design is provided in Table 1.

### 2.4. Behavioural Recordings

Eight cages were filmed simultaneously at 15 frames per second, through the use of eight HD-TVI bullet Infrared cameras (TWE-22MR, Mazi Security Systems GmbH, Tönisvorst, Germany) connected to a digital video recorder (HTVR-0820MT, Mazi Security Systems GmbH, Tönisvorst, Germany). For identification, adult mice were identified by shaving the fur on a 3 cm^2^ area on the left or right thigh in females and the back in males. It was possible to distinguish colours with the lights on, but not with the lights off. Hence, the females’ tail was also coloured using a blue or red sterile surgical grade marker to make the identification more salient during light shifts. Behavioural analyses included detecting the start of parturition, and cases of infanticide or cannibalism, using Microsoft Excel software (v. 16.0, Microsoft Corp., Sacramento, CA, USA).

Video recordings were scanned backwards to determine the exact time when parturition began, i.e., when the first pup was delivered. Once detected, the whole sequence between the start of parturition and next daily cage check was analysed thoroughly by continuous focal sampling to detect pup births and cannibalisms. This method made it possible to correct and validate the number of pups born and to provide an accurate estimate of litter size. The videos were scanned to detect cannibalism each time one or several pups were missing between two daily cage inspections and each time a bitten or partially eaten dead pup was removed. Once cannibalism was detected, the video sequence was rewound to determine the exact start of the event and whether the pup was already dead or actively killed before being cannibalised. The mouse that started the cannibalism (the dam, other female or male) and that killed the pup (when applicable) was recorded, as well as which mice were involved in the cannibalism. When infanticide was detected, details about the context were recorded.

Cannibalism concerned the consumption of the entire pup or only parts of it. When watching videos, cannibalism was considered to take place when an adult performed vigorous head movements while manipulating the pup. The recording was validated by the observation of the partly eaten pup body during its consumption or in the following sequence. Infanticide was defined as actively killing a pup with a series of bites to the head and thorax [27,28]. Infanticide was considered to take place if an adult was seen biting a live pup which was then seen dead. A pup was judged to be alive if it was moving, and dead if it was immobile and stiff when manipulated. It was possible to distinguish skin colour during lights on and this helped to identify whether pups were alive (pink skin) or dead (greyish skin). In some cases, it appeared that the pup was actively killed but the video was not clear enough to confirm this, typically when a newborn pup was cannibalised before it was possible to confirm that it was alive. On one occasion, the pup was seen alive several minutes before being cannibalised but not later. These events were recorded as “probable infanticide”.

### 2.5. Statistical Analysis

Original data used for the analyses are provided as Supplementary Materials Tables S1 and S2. Analyses were carried out using SAS software (v. 9.2, SAS Institute Inc., Cary, NC, USA). For continuous data, the normality of model residuals was tested using the Shapiro–Wilk test of normality. 

To address the first objective, mixed models were used to estimate the effects of inspection method (DAILY_MANUAL vs. VIDEO_TRACK) and litter overlap (TRIO-OVERLAP vs. TRIO-NO_OVERLAP), included as fixed effects, on the number of pups born, died and weaned as well as the risk for complete litter loss. The SOLO litters were excluded since there was no SOLO litters in DAILY_MANUAL cages. Cage identity was included as a random factor to account for clustering and the experimental unit was the litter. For the analysis, the number of dead pups was calculated by subtracting the number of pups at weaning from the number of pups born (estimated as described in detail in Section 2.3. Management Routines). Five litters from TRIO-OVERLAP treatment were born on the same day as the other litter in the cage and it was not possible to discriminate pups from the two litters. In these cases, the total number of newborns were counted and divided by two in order to estimate the focus litter size. The effect of parity and interaction between parity and other factors were tested but not kept in the models since not significant. Data from the number of dead and weaned pups were skewed and Poisson distribution was applied in the models using the Glimmix procedure on SAS. In addition, the effect of counting method on litter loss (binary trait: all pups died; one pup or more survived until weaning) was analysed using mixed logistic regression of the SAS Glimmix procedure. Estimates from models in which transformations were applied were back-transformed to the original scale for presentation in figures and text as predicted means with confidence limits between square brackets. Other estimates were presented as least squares means (± SEM).

The second objective was addressed by using data from the experimental study (VIDEO_TRACK) for which data on date of death and cannibalism were available. The time from birth to pup death detection (at cage check) was analysed using the Kaplan–Meier method and the probability for a dead pup to be cannibalised using mixed logistic regression. Housing (SOLO, TRIO-OVERLAP vs. TRIO-NO_OVERLAP) was included as fixed effects. In the logistic model, cage identity was included as a random factor to account for clustering. The experimental unit was the pup.

To address the third objective, descriptive statistics were used on data from the experimental study (VIDEO_TRACK) to describe whether or not cannibalised pups were killed actively before being cannibalised. Raw data were used for illustration.

### 2.6. Ethical Issues

Although the study of mortality and infanticide raises ethical concerns [19], we studied historical datasets from the breeding management software and pre-recorded video sequences of mouse cages in which pup mortality occurred naturally and unintentionally. Pup mortality was not manipulated through experimental procedures, which were entirely observational in nature. Animal manipulation in this study was limited to gentle handling of females and pups in order to determine the number of alive pups. This is “below-threshold” according to Directive 2010/63/EU Article 1(5f) which defines “practices not likely to cause pain, suffering, distress or lasting harm equivalent to, or higher than, that caused by the introduction of a needle according to good veterinary practice” as exempt from the requirements of the Directive, and not requiring a Project License under United Kingdom’s Animals (Scientific Procedures) Act 1986.

## 3. Results

### 3.1. Litter Size and Mortality Estimates According to the Counting Method (DAILY_CHECK vs. VIDEO_TRACK)

**Figure 2:**
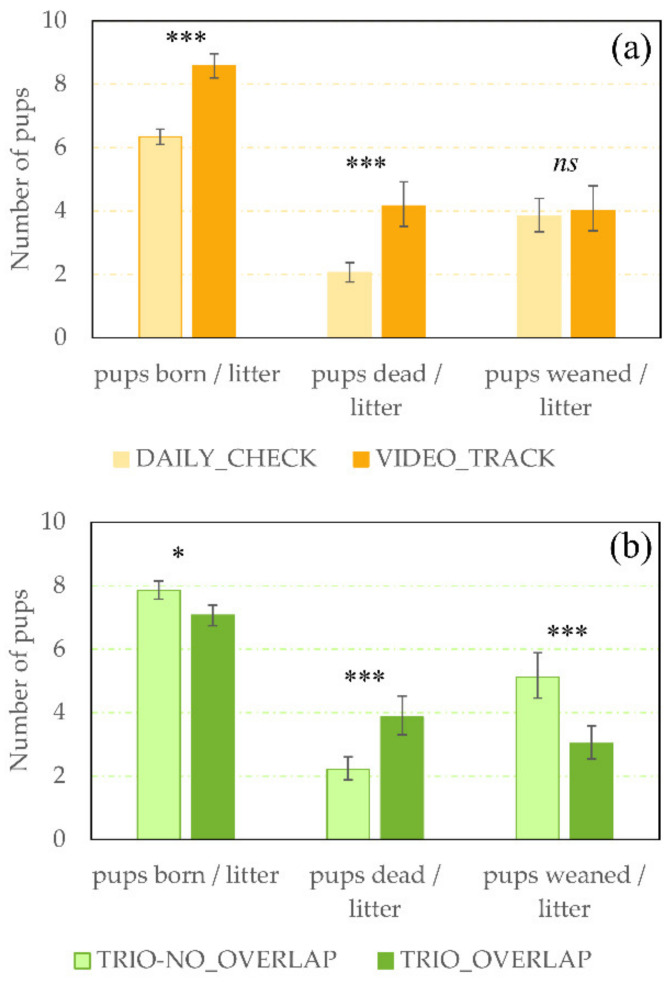
Estimated number of pups per litter according to the counting method and the social configuration. (**a**) Least squares mean (± SEM) number of pups born, predicted mean (CI) number of pups died and predicted mean (CI) number of pups weaned per litter, counted using the daily check counting method without (DAILY_ CHECK, light orange) or with the detailed video analysis of cages between start of parturition and first cage check (VIDEO_TRACK, dark orange), independently of the social configuration. (**b**) Least squares mean (± SEM) number of pups born, predicted mean (CI) number of pups died and predicted mean (CI) number of pups weaned per litter in trio cages without (TRIO-NO_OVERLAP, light green) or with (TRIO-OVERLAP, dark green) the presence of another litter, independently of the counting method. *, ***; Means differ significantly (* *p* < 0.050, *** *p* < 0.001), ns; Means do not differ significantly (*p* > 0.10).

There were no significant effects of the interaction between the counting method and the social configuration, so the interactions were removed from the models and only simple fixed effects were kept and shown. Figure 2a summarises the number of pups estimated using DAILY_ CHECK and VIDEO_TRACK counting methods in the experimental study. For litters where the daily routine counting method was used, the mean litter size recorded was 35% smaller than for the litters where the daily routine counting method was supplemented with video analyses (F_1,43_ = 24.94, *p* < 0.0001). Estimates of total litter loss did not significantly differ between DAILY_ CHECK and VIDEO_TRACK counting (F_1,108_ = 0.26, *p* = 0.613); nor did estimates of the number of weaned pups (F_1,108_ = 0.29, *p* = 0.593). However, the estimation of total number of dead pups per litter differed between counting methods and was more than twice as high in VIDEO_TRACK than in DAILY_ CHECK counting (F_1,108_ = 52.45, *p* < 0.0001).

The Figure 2b shows that the presence of an older litter in the cage at pup birth (i.e., TRIO-OVERLAP litters) affected pup counts by reducing the estimated number of pups counted at birth (F_1,46_ = 3.88, *p* = 0.055), increasing pup loss (F_1,108_ = 30.21, *p* < 0.0001) and reducing number of pups weaned (F_1,108_ = 39.00, *p* < 0.0001). Finally, the risk for complete litter loss (100% of pup mortality) was 1.47% higher for litters from TRIO-OVERLAP than for litters from TRIO-NO_OVERLAP (F_1,108_ = 12.54, *p* = 0.0006).

### 3.2. Timing of Mortality and Cannibalism (in VIDEO_TRACK)

**Figure 3:**
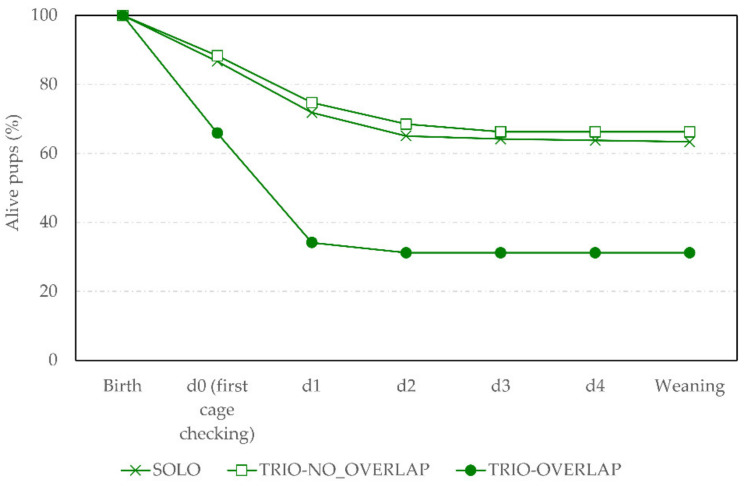
Pup survival across days (d). Survival curve of pups’ survival across days post-birth according to the social housing treatment (SOLO: cross symbol; TRIO-NO_OVERLAP: white square; TRIO-OVERLAP: black circle) in the experimental study (VIDEO_TRACK).

Figure 3 shows the pup survival across days according to the social housing treatment. Higher rates of pup loss were found in TRIO_OVERLAP litters (Kaplan–Meier, $\chi_{2}^{2}$ = 91.82; *p* < 0.0001). Importantly, most of the pup losses happened within the first two days of life, including between birth (i.e., start of parturition) and the first cage check the next morning (i.e., day 0). Pup mortality accounted for pups that were found dead and removed by the first author at the daily cage check, as well as pups that were cannibalised between cage checks.

The probability for a dead pup to be cannibalised by adults differed according to treatment (F_2,372.3_ = 22.37, *p* < 0.0001). Indeed, cannibalism of dead pups was found to be 2.0 and 1.5 times more frequent in TRIO-OVERLAP and TRIO-NO_OVERLAP than in SOLO litters, respectively (predicted means: 61.5%, 46.1% and 21.6% of dead pups cannibalised, respectively, t_389_ = 6.59, *p* < 0.0001 and t_389_ =−4.17, *p* = 0.0001) throughout the 4 days post-birth. In addition, cannibalism of dead pups was found to be 1.4 times more frequent in TRIO-OVERLAP than in TRIO-NO_OVERLAP litters (t_303.9_ = 2.27, *p* = 0.061).

**Figure 4:**
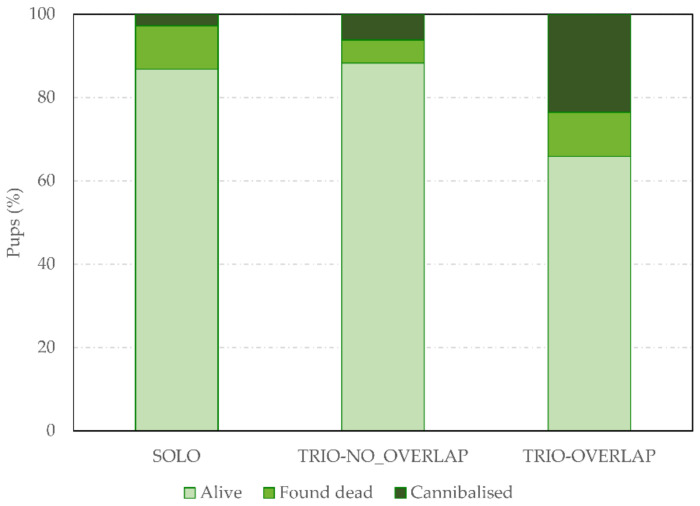
Pup survival at first cage check on day 0. Total percentage of pups that were living, found dead (but not cannibalised) and cannibalised at the first cage check on day 0, according to the social housing treatment (SOLO, TRIO-NO_OVERLAP and TRIO-OVERLAP) in the experimental study (VIDEO_TRACK).

Specifically for the period between birth and the first cage check on day 0, there was also an effect of treatment (F_2,149.3_ = 12.42, *p* < 0.0001). Figure 4 shows the total percentages of pups that were alive, found dead and cannibalised at the time of the first cage check on day 0 for each social housing treatment. Dead pups had 2.5 and 1.7 more risk of being cannibalised when born in TRIO-OVERLAP or TRIO-NO_OVERLAP than in SOLO litters (predicted means: 69.0%, 52.8% and 21.3% of dead pups cannibalised, respectively, t_152_ = 4.94, *p* < 0.0001 and t_149_ = −3.07, *p* = 0.007). At this point in time, there was not significant difference between TRIO-OVERLAP and TRIO-NO_OVERLAP litters. Pups that were cannibalised before the first cage check on day 0 could obviously not be detected by cage checking and were thus detected later using video analyses.

### 3.3. Cannibalism Preceded by Infanticide or Not (in VIDEO_TRACK)

Table 2 summarizes the information related to pup death, including cannibalisms and infanticides. On most of the occasions, pups were visible before and/or at the start of cannibalism (i.e., “Pup visible before and/or at the start of cannibalism”) and it was thus possible to detect whether they were alive and actively killed by the cannibalistic mice or not.

Pups were clearly dead at the start of most of the cannibalism events (i.e., “Not infanticide”). Dams from SOLO litters were never observed actively killing their pups. However, a total of three and four infanticides were clearly detected in TRIO-OVERLAP and TRIO-NO_OVERLAP litters (i.e., “Infanticide”), respectively. In addition, some events could possibly be infanticide (i.e., “Probable infanticide”) but video recordings were not clear enough to confirm this. Details about the infanticides or probable infanticide events are provided in Table 3 and Table 4, and video clips of infanticides and cannibalisms can be shared by the authors on request.

When excluding uncertain events (i.e., “Cannibalism event not detected on video” and “Pup invisible before and/or at the start of cannibalism”), the percentage of pups’ deaths caused by infanticide ranged between 3.4 and 10.1% in TRIO-NO_OVERLAP and between 4.2 and 11.6% in TRIO-OVERLAP litters (probable infanticides are included in the higher number). The infanticides and probable infanticides were always initiated by the dam or the female cagemate. Cannibalism events in trios were also almost always initiated by females (50.4% and 51.2% of cannibalism events initiated by the dam and the female cagemate, respectively) and only three cannibalism events were initiated by the males. Once started, the other female often joined the first one, so that the dam and the female cagemate were involved in 98.4% and 99.2% of the cannibalism events, respectively. In comparison, males participated to a lower extent, and only in 48.8% of the cannibalism events.

## 4. Discussion

Perinatal mortality in laboratory mouse breeding has far-reaching ethical and practical consequences, in that substantial numbers of animals die at a very early age and before they can be used in experiments. We have recently demonstrated that there is large variation in mortality records between facilities for the most commonly used strain, C57BL/6, that mortality rates found in practice are substantially higher than reference data for the strain [5] and that social conditions around birth affect mortality [6]. The present study corroborates and expands on these findings through a detailed analysis of pup cannibalism during the first four days after birth. Our results demonstrate that standard methods for pup counting lead to a dramatic underestimation of perinatal mortality. We furthermore found that infanticide was not a principal cause of death and that most of the cannibalised pups were already dead at the start of the events.

### 4.1. Reliability of the Litter Size and Pup Mortality Estimation Using Daily Check Counting Method

Breeding management practice includes different approaches to counting the number of pups that are born and that die, varying in timing and level of detail in the inspection of periparturient mouse cages. One such approach, chosen with the hope of being practical and reliable, is to check cages once a day and to count pups born on the first day they are detected. In this study, counting pups using this method led to an underestimation of 35% of pups born when compared to data where daily checks were combined with video observations. Continuous video analyses were performed from the start of parturition to the first cage check in order to detect births and cannibalism events that resulted in the disappearance of dead pups before cage checking. Just as litter sizes at birth were underestimated, the total number of dead pups was also underestimated by using daily check counting. More specifically, the estimation of the number of dead pups was twice as low using daily check counting than after validation through video analyses. Taken together, these results suggest that in practice, a considerable proportion of pups that die and disappear are never registered as being born.

The underestimation of the number of pups born may be related to the method used at the daily cage check. In the experimental study, the researcher counted pups once a day, between day 0 and 4, after opening the cages systematically at the change station. By contrast, lab technicians opened cages at day 0 only when dead pups were detected or when the litter was not clearly visible through the translucent cage wall. Counting pups through the cage wall could lead to an underestimation of the number of pups present in the cage since individual pups hidden in the middle of the nest could be missed. However, it is important to note that all technicians responsible for pup counting in this study were highly skilled and trained and human counting errors are unlikely to be sufficient to explain the large discrepancy between data from the two methods. It is also possible that the more detailed method where cages were opened several times and pups touched increased pup mortality by causing more nest disturbance. However, this would also be expected to lead to fewer pups weaned. In this study, the number of pups weaned did not differ between the two counting methods. Although the authors agree that unnecessary disturbance should be avoided, this finding indicates that there was no decrease in breeding performance with the more thorough counting method compared to the daily counting. In line with this, Peters et al. [29] showed that conducting close daily inspections (i.e., involving removing the cage lid and, if necessary, nesting material) was unlikely to increase inbred BALB/cOlaHsd pup mortality since breeding performance did not differ from that of low-disturbance daily inspection (i.e., cage not opened). We therefore suggest that the different results produced by the different pup-counting methods result primarily from the occurrence of cannibalism between birth and the subsequent first cage check.

The observation that litters born in cages where another litter was already present at the time of birth (i.e., TRIO-OVERLAP) were more likely to die than litters born in cages without another litter at the time of birth (i.e., TRIO-NO_OVERLAP) had already been reported in the first paper from this study, Brajon et al. [6]. This was independent of pup-counting method. In a large retrospective study of nearly 20,000 litters, Morello et al. [5] demonstrated that the probability of pup death increased with the number and age of older siblings. Because there is no reference age gap beyond which the risk to die suddenly increases (this is a gradual effect that is intimately related to other factors such as the number of sibling pups), for the present analysis we decided to include all litters born while another litter was present in the cage for TRIO-OVERLAP modality, independently of the age gap. However, based on our previous work [5], we acknowledge that the effect size in the models may be higher if only litters with a larger age gap (e.g., more than 8 days difference between focus and sibling litter) were considered in TRIO-OVERLAP litters. The impact of the presence, age and number of sibling litters in the cage around newborn birth is an inherent problem in breeding systems that permanently pair a male with two females, but this can also occur in cages where a male is paired with a single female, especially when sibling litters are weaned a little late, beyond 21 days.

### 4.2. Cannibalism as a Cause of Pup Mortality Underestimation

Results from the video observations in combination with cage checks indicate that pup deaths occurred mainly within the first two days of life, and so before the second cage check on day 1. The probability for a pup to die within the first 48 h post-birth was about twice as high when another litter was already present in the cage at birth, as already showed in Brajon et al. [6]. Pup mortality was also high within the first day of life, as found in previous studies [11,26]. Indeed, in trio cages with litter overlap, 34.1% of pups died before the first cage check on day 0, whereas the same figures for trios without litter overlap and solo were 11.7% and 13.0% pups, respectively.

Cannibalism is part of the mouse behavioural repertoire [30] and was frequent in this study. Amongst dead pups, the proportion that were also cannibalised was evaluated. Dead pups were more likely to be cannibalised in trio cages, where three adult mice were present, compared to solo cages where the dam was the only adult. Detailed analyses of cannibalism events in trios indicated that the cannibalism initiator was almost always either the dam or the female cagemate, and the second female almost always joined the initiator to consume the dead pup for most of the duration of the event. In comparison, cannibalism was rarely initiated by males, and they joined the initiator to eat the dead pup in less than 50% of the cannibalism events, and for a shorter duration. Among trios, dead pups from trios with litter overlap were more likely to be cannibalised than dead pups from trios without litter overlap, throughout the four days post-birth.

Such high rates of cannibalism make very difficult to reliably establish mortality rates without the aid of video recordings. A total of 21.3% of dead pups on day 0 were already cannibalised before the first cage check in litters born in SOLO cages with a single mother, and this reached 52.8% and 69.0% of dead pups cannibalised in litters from trios without and with litter overlap, respectively. This means that more than half of the pups that died on day 0 had already disappeared at the time of the first cage check and could never be detected by this method alone. As a proportion of all pup deaths up to day four, this corresponds to a total of 18.3% (19/104 deaths) and 34.2% (40/117 deaths) of the dead pups that were cannibalised before being counted in trios without or with litter overlap. We are therefore confident that the differences in litter size at birth and in mortality between data from the experimental study and data from the breeding colony during the same period are largely the result of a high rate of previously undetected cannibalisms.

Both birth and mortality rates may also be generally underestimated in other breeding facilities. In the present paper, we demonstrate the extent to which checking cages on a daily basis and counting pups as soon as they are detected on day 0 under-reports the number of pups that are born. While comprehensive data from across facilities are missing, results of a preliminary survey of breeding facilities in Europe show that only 3/20 surveyed facilities in Europe declared daily cage checking to detect and count pups on the day of birth (unpublished data). A more common practice (13/20 breeding facilities) was simply to count pups when discovered at cage cleaning during the first week post-birth. Three other breeding facilities counted pups one week after birth or later, and a last breeding facility reported not counting pups before weaning. The pups that we have observed to die and be cannibalised within the first two days of life would be mostly undetected using these methods. The authors believe that many mouse breeding facilities might be unaware of their true perinatal mortality rate; this ethical issue is increasingly debated and contested [31]. While pups found dead on daily checks were removed from the cage in this study, future studies should explore the rates of cannibalism when dead pups are not removed across multiple days to properly estimate the proportion of cannibalised (and undetected) dead pups when checking cages for the first time two days after birth or later.

### 4.3. Infanticide or Cannibalism?

Detailed video analyses allowed us to detect whether pups were already dead at the start of the cannibalism event (cannibalism only) or were actively killed by an adult (infanticide followed by cannibalism). In our detailed video recordings from 109 litters and 950 individual pup births, infanticide was clearly detected on only seven occasions, with an additional 13 probable infanticides, including 12 events that happened within a few minutes of the pup’s birth, meaning that it was not possible to know whether the pup was born dead or actively killed at birth. All of these events happened in trios. Infanticide is often put forward as a cause of pup death and has been extensively described in experimental studies with wild house mice [23], although Perrigo et al. [32] reported that domesticated CF-1 dams were less likely to be infanticidal than wild stock house mice. It is however problematic that the criteria for infanticide are often missing or are conflated with cannibalism, including in several studies from the behavioural literature where infanticide was inferred from the disappearance of pups between two cage checks [18,24]. Rather than infanticide, pup disappearance could be caused by cannibalism after death for other reasons than infanticide. Hauschka [33] reported that detailed and frequent inspections of laboratory mouse pups allowed him to separate death from congenital causes from death caused by the parents. However, contemporary studies reporting infanticide in laboratory mice have not used sufficiently clear criteria in combination with detailed enough behavioural observation throughout the 24 h post-parturition to reliably demonstrate that pups were actively killed.

Overall, our data suggest that pup infanticide is infrequent under normal conditions for laboratory mouse breeding, but it still happens. In the first detailed behavioural study of this topic in C57BL/6 mice, Weber et al. [26] found only cannibalism of pups that were already dead, and failed to detect any infanticide. However, with only ten females and video recordings encompassing only 15 min per h, this study may have failed to detect active pup killing due to the small animal sample size and gaps in video sequence analysis. In addition, mice were single-housed and our data show that infanticide is more probable when a female cagemate is present. Communal nesting is frequent in rodents, including in the laboratory mouse, and cooperation for breeding provides benefits such as shared caring effort, enhanced thermoregulation and group defence against intruders [34,35,36], but also creates opportunities for cheating and exploitation by female cagemates [37,38,39]. Infanticide of congeners’ pups in this context is an adaptive behaviour that allows a female to manipulate the relative contribution to the communal litter in her own favour and thus increase her reproductive success. One risk factor for infanticidal conflict is asynchronous breeding [40], when a litter is born after the other one, such as in most of the litter overlap cages in our data. In wild house mice (*Mus domesticus*), it is typically the pregnant female who kills existing pups of the other female before she gives birth herself [23,25,41]. This may explain infanticides or probable infanticides by gestating female cagemates at pup birth in our study. However, some infanticides were caused by the dam herself. In one occasion, a pup was actively killed through infanticide by the dam with dystocia, characterised by difficulty to deliver pups [42]. This female was culled 48 h after the start of parturition on welfare grounds. Savaging toward own newborn is well-known in pigs and has been found to be more frequent in gilts [43,44], suggesting that maternal experience may mitigate infanticide behaviours. Maternal infanticide has also been suggested as a behavioural strategy in response to unusually large or small litter size, reduced food supply or disturbance [19,45]. However, the underlying causes of pup infanticide are not always understood since both physiological state and environmental factors may interfere and motivate mice to actively kill newborns [25,30,46]. No male was seen killing a pup. Well-established males in a group are not infanticidal toward offspring produced by female partners [27,28,47] and thus, this conflict mainly concerns females. Our study is the first to video track and directly detect infanticidal behaviours in females under standard laboratory breeding conditions.

## 5. Conclusions

The results presented in this paper show that once-daily cage inspections may result in a substantial underestimation of the number of pups born, as a result of high numbers of pups that die and are cannibalised by adults before the first cage inspections. If these findings are representative of breeding practices, many breeding facilities are likely to be unaware of their true mouse pup mortality. There is no support for the popular belief that infanticide by the females is an important cause of perinatal mortality. Paradoxically, the common practice of not inspecting cages around parturition, not only conceals the problem of perinatal mortality but also inhibits a better understanding of the causes of pup death. As an important first step towards addressing the problem of mortality, we recommend that breeding facilities inspect breeding cages daily after parturition, including on the first day. Based on our current knowledge of risk factors, a research priority should be to develop strategies minimising the occurrence of litter overlap.

## Figures and Tables

**Table 1:** Summary of the study design.

	Breeding Facility Data	Experimental Study Data
Selection criteria	C57BL/6Babr, Breeding room, Parities 2–5, March–June 2017	C57BL/6Babr, the same breeding room, Parities 2–5, March–June 2017, different litters
Social configuration	Trio-overlap (70) Trio-no_overlap (68)	Trio-overlap (20) Trio-no_overlap (35) Solo (* originally housed in trio, 54)
Data collection	Data extracted from the breeding facility management software. Counting method described in Figure 1	Data collected by the first author of the paper. Counting method described in Figure 1
Litter size estimation	Number of pups born and dead counted through the cage wall or at the change station at day 0, if needed (first detection)	Number of pups born and dead counted at the change station at day 0 (first detection) + number of pups cannibalised between birth and day 0
Name of the counting method	DAILY_CHECK	VIDEO_TRACK
Data used for obj. 1: Comparing litter size and mortality estimates according to counting method	Yes	Yes, but only litters from trios
Data used for obj. 2: Evaluating timing of pup mortality and cannibalism	No	Yes
Data used for obj. 3: Determining whether cannibalisms were preceded by infanticide or not	No	Yes

**Table 2:** Summary information about pup mortality until day 4 in the experimental study (VIDEO_TRACK).

Number of Pups	SOLO	TRIO-NO_OVERLAP	TRIO-OVERLAP
Total born	472	308	170
Total died until day 4	171	104	117
Found dead			
Day 0 Day 1 Day 2 Day 3 Day 4 Total	48 56 26 3 1 134	17 21 13 5 0 56	18 26 1 0 0 45
Cannibalised			
Day 0 Day 1 Day 2 Day 3 Day 4 Total ^a^	13 15 6 1 2 37	19 21 6 2 0 48	40 28 4 0 0 72
	(21.6% of dead pups)	(46.1% of dead pups)	(61.5% of Dead pups)
Cannibalism events not detected on video ^b^	5	1	4
Pup invisible before and/or at the start of cannibalism ^c^	4	14	18
Pup visible before and/or at the start of cannibalism ^d^	28	33	50
Not infanticide	28	24	39
Infanticide	0	3	4
Probable infanticide	0	6	7
Infanticide range	0	3–9	4–11

^a^ The percentages of cannibalised pups between parentheses correspond to the ratio between the total number of pups cannibalised divided by the total number of pups found dead until day 4. ^b^ Pup missing between two cage checks but cannibalism events not detected on video analyses. ^c^ Cannibalism events detected but pup invisible before and at the start of the cannibalism event, preventing from seeing whether the pup was alive or not at the start of the cannibalism event. ^d^ Pup visible before and/or at the start of cannibalism and it was thus possible to detect whether it was dead at the start of cannibalism event (i.e., “Not infanticide”) or alive and actively killed by the cannibalistic mice (i.e., “Infanticide”). On certain occasions, some cues suggested that the pup was actively killed but the video sequence was not clear enough to confirm this (i.e., “Probable infanticide”). The infanticide range indicate the minimum (i.e., “Infanticide” only) and the maximum (i.e., “Infanticide” + “Probable infanticide”) number of plausible infanticides.

**Table 3:** Summary information about pup infanticide detected in litters born from trio cages without litter overlap (TRIO-NO_OVERLAP) in the experimental study (VIDEO_TRACK).

Litter	Nb of Pups Born	Nb of Pups Weaned	Parity	Infanticide	Infanticidal Mouse	Time after Pup Birth	Comments
L_A_	8	7	3	Yes	Female cagemate	At birth	Gives birth to her own litter 21 h after the infanticide
L_B_	8	0	5	Yes	Dam	<4 h after birth	Parturition issues (dystocia), the dam was culled 48 h after the start of the parturition
L_C_	9	7	3	Yes	Dam	<4 h after birth	Pup seen moving but with a blue colour skin before infanticide
L_D_	8	0	4	Probable	Dam	At birth	Eaten as soon as born but not seen moving, perhaps born dead
				Probable	Dam	<4 h after birth	It seems that all pups were visible and alive before the start of the event, but does not seem to kill (no vigorous head movements)
L_E_	12	10	4	Probable	Dam or Female cagemate	At birth	Eaten as soon as born but not seen moving, perhaps born dead. The two females handled and ate the pup at the start of the event.
L_F_	8	7	4	Probable	Female cagemate	At birth	Eaten some minutes after birth but not seen moving, perhaps born dead
L_G_	9	4	3	Probable	Female cagemate	At birth	Eaten as soon as born but not seen moving, perhaps born dead
L_H_	5	0	3	Probable	Female cagemate	At birth	Eaten as soon as born but not seen moving, perhaps born dead

**Table 4:** Summary information about pup infanticide detected in litters born from trio cages with litter overlap (TRIO-OVERLAP) in the experimental study (VIDEO_TRACK).

Litter	Nb of Pups Born	Nb of Pups Weaned	Parity	Infanticide	Infanticidal Mouse	Time after Pup Birth	Comments
LI	8	6	3	Yes	Female cagemate	<4 h after birth	Gives birth to her own litter 4 h after the infanticide
LJ	9	0	4	Yes	Female cagemate	At birth	Gives birth to her own litter 3 to 6 days later
				Probable	Female cagemate	At birth	Gives birth to her own litter 3 to 6 days later, eaten as soon as born but not seen moving, perhaps born dead
				Probable	Female cagemate	At birth	Gives birth to her own litter 3 to 6 days later, eaten as soon as born but not seen moving, perhaps born dead
				Probable	Female cagemate	<4 h after birth	Gives birth to her own litter 3 to 6 days later, eaten after completing previous cannibalisms. The pup was alive the last time it was seen but invisible at the start of the event.
LK	11	0	4	Yes	Dam	<24 h after birth	No specific observation.
				Probable	Dam or Female cagemate	At birth	Eaten as soon as born but not seen moving, perhaps born dead
LL	10	0	3	Yes	Female cagemate	At birth	No specific observation
				Probable	Female cagemate	At birth	Eaten as soon as born but not seen moving, perhaps born dead
LM	10	6	2	Probable	Female cagemate	At birth	Eaten as soon as born but not seen moving, perhaps born dead
LN	8	0	3	Probable	Dam	At birth	Eaten as soon as born but not seen moving, perhaps born dead

## Data Availability

Datasets are available at the supplementary materials, Table S1. Dataset of the DAILY_CHECK and VIDEO_TRACK litters used for the objective 1; Table S2. Dataset of the VIDEO_TRACK pups used for the objectives 2 and 3.

## Supplementary Materials

The following are available online at https://www.mdpi.com/article/10.3390/ani11082327/s1, Table S1. Dataset of the DAILY_CHECK and VIDEO_TRACK litters used for the objective 1; Table S2. Dataset of the VIDEO_TRACK pups used for the objectives 2 and 3.
